# Identifying the Role of Spatial Transitions in the Palliative Care Experiences of Unhoused Older Adults

**DOI:** 10.1093/geroni/igaa057.817

**Published:** 2020-12-16

**Authors:** Ian Johnson, Michael Light

**Affiliations:** 1 University of Washington, Seattle, Washington, United States; 2 Harborview Medicine, Seattle, Washington, United States

## Abstract

The national population of unhoused older adults is predicted to nearly triple by 2030. Unhoused older adults have a mortality rate four to nine times higher than housed populations, and face structural barriers during illness trajectory that likely influence both where care takes place and processes around attaining psychosocial later-life goals. This study aimed to (1) document patterns in the healthcare trajectories of unhoused older adults and (2) examine the role of care transitions in psychosocial goal attainment. Retrospective chart review was completed in partnership with a mobile homeless palliative care team; additional data was gathered from provider focus group. Through a content analysis of this data, it was discovered that older unhoused palliative care patients experienced more transitions, and that numerous care transitions were associated with disruptions to goals. The type of care transitions patients experienced also did not reflect their later-life goals. Patients’ movements impacted the role of formal and informal care networks in care and highlighted the implications of place in common psychosocial goals, such as family reconciliation. Unconventional neighborhood supports were found to help facilitate treatment adherence. These findings offer translational opportunities for further research, including “rapid respite” models and other innovations in mobile palliative care for unhoused and precariously-housed people with life-limiting illness.

